# Quadrupole lattice resonances in plasmonic crystal excited by cylindrical vector beams

**DOI:** 10.1038/srep34967

**Published:** 2016-10-13

**Authors:** Kyosuke Sakai, Kensuke Nomura, Takeaki Yamamoto, Tatsuya Omura, Keiji Sasaki

**Affiliations:** 1 Research Institute for Electronic Science, Hokkaido University, Sapporo, Hokkaido, 001-0020, Japan

## Abstract

We report a scheme to exploit low radiative loss plasmonic resonance by combining a dark (subradiant) mode and a lattice resonance. We theoretically demonstrate that such dark-mode lattice resonances in periodic arrays of nanodisks or plasmonic crystals can be excited by vertically incident light beams. We investigate the excitation of lattice resonances in a finite sized, square-lattice plasmonic crystal by two types of cylindrical vector beams and a linearly polarized Gaussian beam. Quadrupole lattice resonances are excited by all three beams, and the largest peak intensity is obtained by using a specific type of cylindrical vector beam. Because of their lower radiative losses with many hotspots, the quadrupole lattice resonances in plasmonic crystal may pave the way for photonic research and applications that require strong light-matter interactions.

Localized surface plasmon resonances (LSPRs) on metal nanoparticles (NPs) can concentrate light into nanoscale volumes, thereby strongly enhancing the electromagnetic field. Applications that use this unique property of LSPRs include the control of light-matter interactions^1^,^2^, chemical or bio-sensors^3^,^4^, surface-enhanced Raman spectroscopy^5^, photovoltaic cells^6^, photocatalysts^7^, and nano-lasers^8^. However, plasmon damping or losses prevent the further enhancement of the localized electromagnetic field and thus represent one of the main barriers to the evolution of plasmonics into a prominent technology. It should be possible to control damping caused by resistive ohmic heating inside the metal by controlling the properties of the material^9^, whereas radiative losses (the decay of plasmons into photons) can be managed by the following two approaches.

The first approach is to exploit the dark (subradiant) mode of an LSPR by using strategies such as the symmetry breaking of nanoparticles^10^,^11^, phase retardation of the incident radiation across the particle^12^, interaction with the dipole modes in coupled particles^13^, and interference between dark and bright modes, i.e., electromagnetically induced transparency^14^ or Fano resonances^15^. We have recently reported a method of exploiting subradiant multipole LSPRs by using vortex beams with specific angular momentum^16^.

The second approach to managing radiative losses is to take advantage of either the lattice resonances in periodic NPs or, in other words, the photonic band-edge effect of plasmonic crystals, in which collective resonances are mediated by the diffractive coupling of localized plasmons. Sharp spectral line shapes have been reported by several groups on the basis of both theoretical and experimental work^17^,^18^,^19^,^20^,^21^,^22^,^23^. The dispersive properties of the lattice plasmon and the tunability of the line shape have also been reported^24^,^25^.

In this letter, we present a combination of the two above approaches applied to the management of radiative losses, taking advantage of multipolar dark lattice resonances, which can be excited from free space. We theoretically demonstrate that the quadrupole lattice resonances in square lattice plasmonic crystals composed of nanodisks can be excited both by vertically incident cylindrical vector (CV) beams and by a linearly polarized Gaussian beam. This idea was inspired by observations that cylindrical vector beams with radial or azimuthal polarization are emitted from the antisymmetric (subradiant) band-edge modes of a photonic crystal laser^26^,^27^. Despite the destructive interference of these antisymmetric band-edge modes, the laser output can be coupled to free space as surface emission, owing to the finite size effect of the laser cavity^28^. Here, we use the inverse process, in which an incident light beam from free space couples to the antisymmetric lattice modes of the plasmonic crystal. We first perform eigenmode analysis in an infinitely periodic system to elucidate which type of lattice resonances should be obtained. Second, we apply different vertically incident beams to a finite system to investigate which lattice resonances are excited by the beams. Our findings are not limited to quadrupole resonances but can be extended to other multipole lattice resonances, such as the hexapole resonance in a triangular lattice crystal.

## Results

### Calculation model and eigenmodes in infinite system

**Figure 1:**
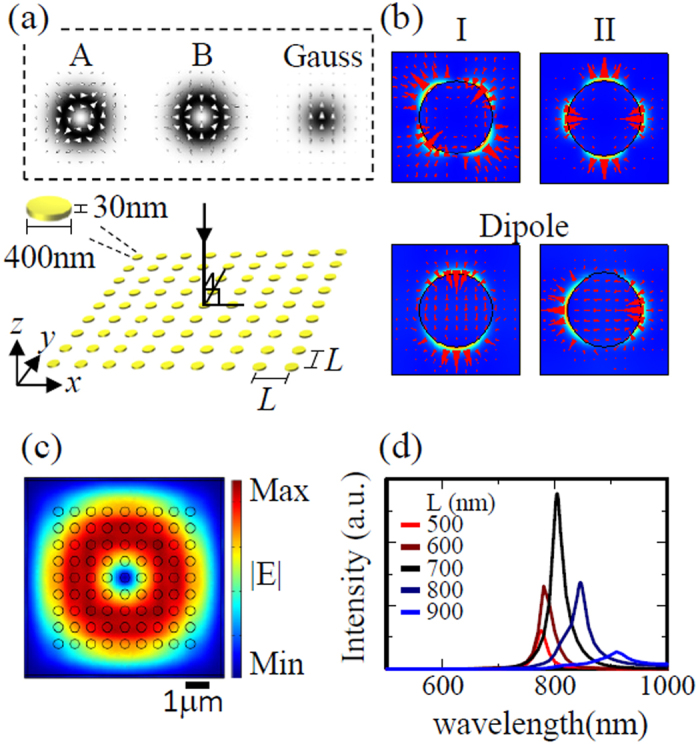
(**a**) Calculation model: a square-lattice plasmonic crystal consisting of gold nanodisks. The inset shows the cross-sections of three incident beams: cylindrical vector beams A and B and a linearly polarized Gaussian beam. The arrows indicate the electric field vectors. (**b**) Electric field distributions of the four eigenmodes in the unit cell of an infinite periodic system with a lattice period L = 700 nm. The triangles indicate the electric field vectors. (**c**) Electric field norm profile of incident cylindrical vector beam A in free space. The black circles indicate the positions of nanodisks for the crystal with L = 700 nm. (**d**) Dependence of near-field electric field intensity on lattice period for a finite periodic system (9 × 9 nanodisks).

Figure 1(a) shows the model used in our calculations. For simplicity, we consider a square lattice plasmonic crystal in a homogeneous background with a refractive index of n = 1.0. The structure consists of gold nanodisks with a diameter of 400 nm and a thickness of 30 nm, which yields LSPR peaks at approximately 800 nm. The lattice period (L) is chosen such that normally incident light along the -z direction is diffracted in the x or y directions (first-order grating). The optical constant of gold was taken from Johnson and Christy^29^.

To obtain a basic understanding of the lattice resonances that are excited, we performed eigenvalue calculations by using an infinite periodic system with Bloch boundary conditions in the x and y directions for a period (L) of 700 nm. Figure 1(b) shows the electric field distributions of the four eigenmodes within the unit cell, which we assume to be the band-edge modes of the photonic band structure at the Gamma-point^27^,^28^. We obtained two types of quadrupole modes (I at 820 nm, II at 715 nm) and doubly degenerate dipole modes at 1045 nm. In an infinite periodic system, the two quadrupole modes do not couple to the far field, owing to the antisymmetric nature of the electric field (dark modes), whereas they can couple to the far field in a finite system^28^. In contrast, light coming from free space can couple to the two quadrupole modes in a finite system. For efficient coupling, the symmetries of the incoming light and of the quadrupole modes should match.

### Lattice resonances in finite system excited by normally incident light beams

To simulate the finite case, we used a plasmonic crystal consisting of a 9 × 9 array of nanodisks, which was the largest feasible size for our computer resource. Two types of CV beams (A, B) and a linearly polarized Gaussian beam, shown in the inset of Fig. 1(a), were chosen to excite different lattice resonances. The polarization of CV beams A and B is linear at all points, although the direction of the electric field vector depends on the spatial position^30^. Figure 1(c) shows the cross-sectional intensity profile of the incident CV beam A in free space. The black circles indicate the positions of the nanodisks comprising the plasmonic crystal with a period of 700 nm, for which the whole crystal area is covered by the incident beam. Figure 1(d) shows the near-field spectrum of mode I excited by CV beam A as a function of the lattice period. The spectrum for L = 700 nm shows a maximum peak intensity at a wavelength of approximately 800 nm, which corresponds to the peak wavelength of the LSPR of a single isolated nanodisk. We therefore set the lattice period to 700 nm in the subsequent analysis.

To understand which lattice resonances can be excited by each type of incident beam, we calculated the electric field intensity spectrum integrated over the x-y plane of the crystal, as well as the near-field distribution at the peak wavelength of the integrated spectrum. We also calculated the normalized near-field intensity spectra at the points where the respective lattice resonances are strongly confined. All calculations were performed on the plane at half the disk height.

**Figure 2:**
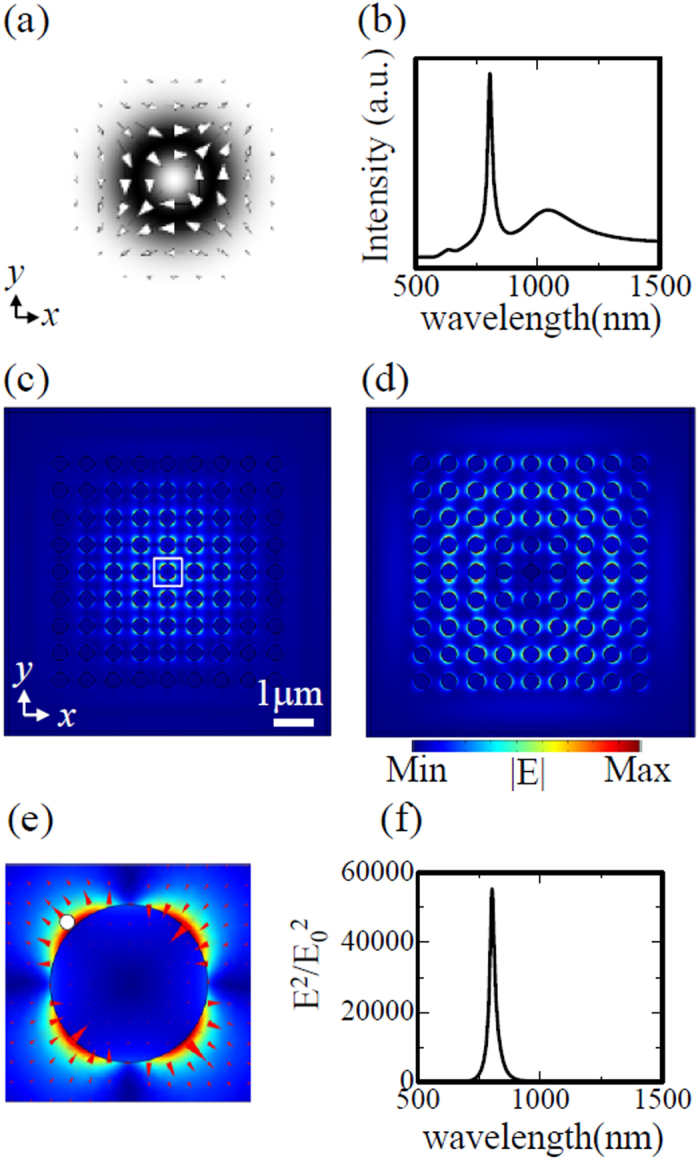
(**a**) Cross-sectional profile of incident cylindrical beam A. The arrows indicate the electric field vectors, and the grey shading indicates the electric field norm distribution. (**b**) Electric field intensity spectrum integrated over the x-y plane of the crystal, with peaks at 805 nm and 1040 nm. (**c**) Electric field norm distribution at a wavelength of 805 nm. (**d**) Electric field norm distribution at a wavelength of 1040 nm. (**e**) Detailed view of electric field norm distribution inside the white box in (**c**). The triangles indicate the electric field vectors. (**f**) Normalized near-field intensity spectrum at the hot spot indicated by the white dot in (**e**).

When CV beam A, shown in Fig. 2(a), is used as the excitation beam, the near-field electric field intensity (|E|^2^) spectrum integrated over the x-y plane exhibits two clear peaks, as shown in Fig. 2(b): a narrow peak at 805 nm (quadrupole mode I) and a broad peak at 1040 nm (dipole mode). Figure 2(c) shows the near-field norm (|E|) distribution at 805 nm, in which the single-lobed envelope function peaks at the centre, even though the incident beam has a ring-shaped intensity profile. In finite-sized crystals, the envelope function is known to have both fundamental and higher-order modes^27^. Figure 2(c) indicates that CV beam A excites the fundamental mode of the envelope function. In contrast, the near-field distribution at 1040 nm shown in Fig. 2(d) exhibits a ring-shaped envelope function corresponding to a higher-order mode, with a dipole-like intensity profile on each nanodisk. The axis of the dipole on each disk is parallel to the electric field vector of the incident beam, as shown in Fig. 2(a). Figure 2(e) shows a detailed view of the electric field profile around the central nanodisk of Fig. 2(c), thus indicating that this 805 nm lattice resonance corresponds to quadrupole mode I in Fig. 1(b). The near-field intensity (|E|^2^/|E_0_|^2^) spectrum in the hot spot situated 1 nm from the nanodisk sidewall, indicated by the white dot in Fig. 2(e), possesses a narrow line shape, as shown in Fig. 2(f). The full width at half maximum (FWHM) is ~30 nm, which is much narrower than the FWHM of ~80 nm for a single isolated nanodisk. This result indicates that the lattice resonance decreases the radiative loss even in a finite crystal with a 9 × 9 nanodisk array. The peak intensity reaches a very large value of more than 5 × 10^4^. We note that, the intensity (|E|^2^) was normalized by the ring-shaped incident beam intensity obtained without nanodisks (|E_0_|^2^), thus causing the normalized intensity to reach a relatively large value near the centre. Nevertheless, the field enhancement of quadrupole mode I is expected to be sufficient to induce strong light-matter interactions.

**Figure 3:**
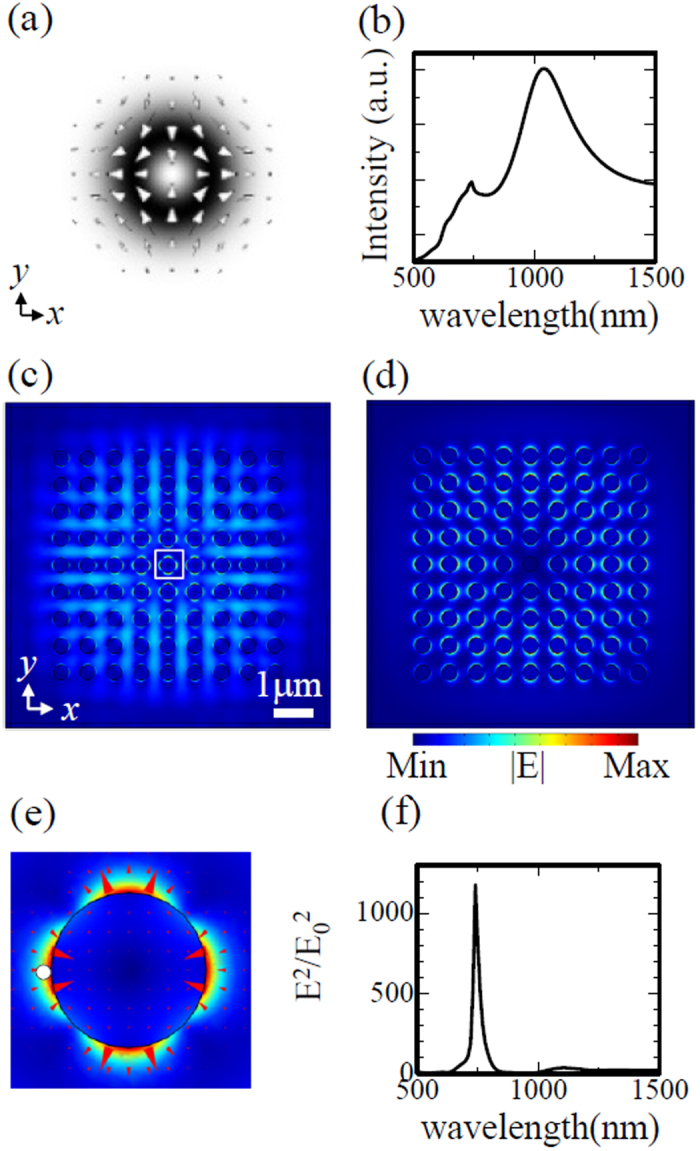
(**a**) Cross-sectional profile of incident cylindrical beam B. The arrows indicate the electric field vectors, and the grey shading indicates the electric field norm distribution. (**b**) Electric field intensity spectrum integrated over the x-y plane of the crystal with peaks at 740 nm and 1040 nm. (**c**) Electric field norm distribution at a wavelength of 740 nm. (**d**) Electric field norm distribution at a wavelength of 1040 nm. (**e**) Detailed view of electric field norm distribution inside the white box in (**c**). The triangles indicate the electric field vectors. (**f**) Normalized near-field intensity spectrum in the hot spot indicated by the white dot in (**e**).

If CV beam B is used as the incident beam instead of A, corresponding to a 45 degree rotation of the beam cross-section with respect to the beam centre, the excited quadrupole lattice resonance is mode II. The integrated near-field intensity in Fig. 3(b) shows a small peak at 740 nm for mode II and a large peak at 1040 nm for the dipole mode. The near-field distribution at 740 nm in Fig. 3(c) shows distinct quadrupole profiles around the centre. Because the incident beam partially passes through the crystal, the ring-like intensity pattern is also observed between the nanodisks in Fig. 3(c). The near-field distribution at 1040 nm in Fig. 3(d) takes the form of a ring-like envelope function with a dipole intensity profile on each nanodisk. The axis of the dipole on each disk is parallel to the electric field vector of the incident beam, as shown in Fig. 3(a). Figure 3(e) shows the detailed electric field profile around the central nanodisk in Fig. 3(c), indicating that the lattice resonance excited around the central region corresponds to quadrupole mode II. The near-field intensity spectrum at the point indicated by the white dot in Fig. 3(e) exhibits a narrow line shape with a FWHM of ~30 nm, as shown in Fig. 3(f). However, the peak intensity is smaller than that for quadrupole mode I, owing to the lower coupling efficiency from the incident beam.

**Figure 4:**
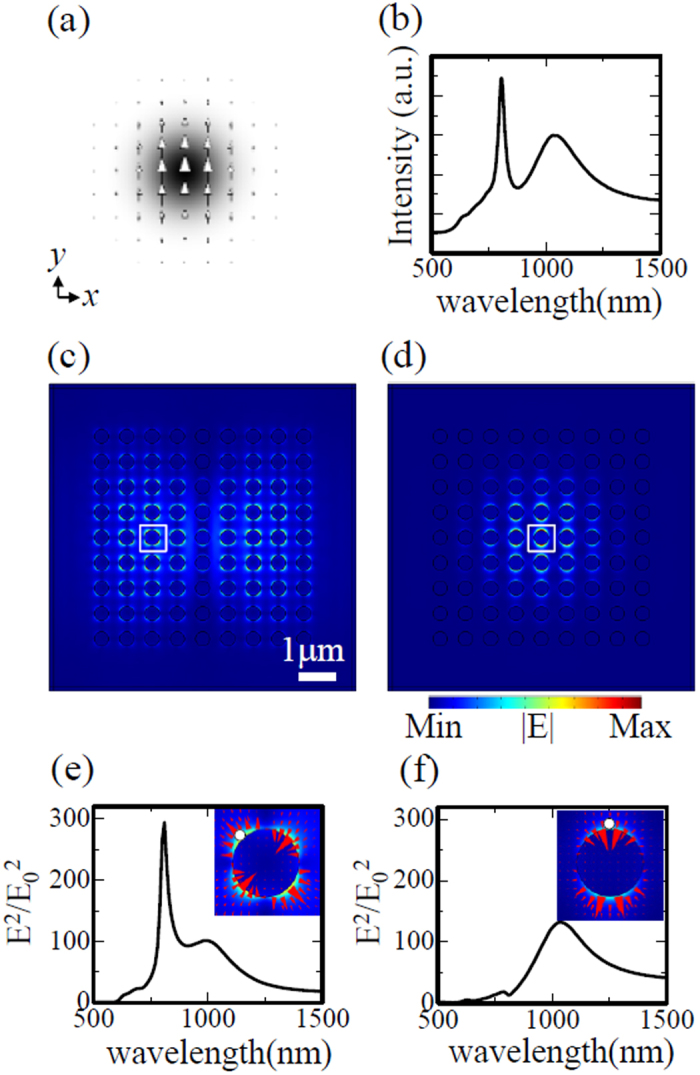
(**a**) Cross-sectional profile of linearly polarized Gaussian incident beam. The arrows indicate the electric field vectors, and the grey shading indicates the electric field norm distribution. (**b**) Electric field intensity spectrum integrated over the x-y plane of the crystal, with peaks at 805 nm and 1040 nm. (**c**) Electric field norm distribution at a wavelength of 805 nm. (**d**) Electric field norm distribution at a wavelength of 1040 nm. (**e**) Normalized near-field intensity spectrum in the hot spot indicated in the inset. The inset shows a detailed view of the near-field profile inside the white box in (**c**). (**f**) Normalized near-field intensity spectrum in the hot spot indicated in the inset. The inset shows a detailed view of the near-field profile inside the white box in (**d**).

The quadrupole lattice resonance is excited even using a Gaussian incident beam. Figure 4(b) shows the corresponding integrated intensity spectrum, in which there are two clear peaks: a narrow peak at 805 nm and a broad peak at 1040 nm. The near-field distribution at 805 nm in Fig. 4(c) shows a double-lobed higher-order envelope function along the x axis with a quadrupole profile around the nanodisks. Although it is not apparent from the figure, the phases of the left and right-hand parts are π shifted with respect to each other. As a result, the quadrupole mode is cancelled out, and only a small dipole mode can be observed in the central column. The detailed field distribution around the nanodisk indicated by the white square corresponds to quadrupole mode I, as shown in the inset of Fig. 4(e). The normalized near-field intensity spectrum shows both a sharp quadrupole peak and a broad dipole peak, thus indicating that the two modes overlap each other in space and frequency. At 1040 nm, the near-field distribution exhibits dipole lattice resonances with a single-lobed fundamental envelope function, as shown in Fig. 4(d). The normalized near-field intensity spectrum at the central nanodisk comprises a dipole peak and a small quadrupole peak, as shown in Fig. 4(f). Notably, all of the dipole resonances obtained in this study possess similar near-field spectra with a peak wavelength of ~1040 nm, indicating that they all originate from the dipole lattice resonance in the infinite crystal shown in Fig. 1(b), though there are considerable differences in the envelope functions of the field distributions. Through comparison of the spectral line widths of the dipole and quadrupole lattice resonances, it is apparent that the quadrupole resonance has much lower radiative loss.

## Discussion

We demonstrated the excitation of quadrupole lattice resonances with a model comprising a specific size of crystal, i.e., a 9 × 9 array, owing to limited computer resources. However, our findings can be applied to smaller or larger crystals. In small-scale crystals, the near-field spectra and the field distributions demonstrate the excitation of the quadrupole lattice resonance, even in a 3 × 3 array, and the trend in the spectral line width shows converging behaviour as the array size increases (Supplementary Note 1). We assume that the area where the quadrupole lattice resonances are excited should be defined by the area irradiated by the incident beams. In the present work, we set the incident beam size to nearly the same size as the 9 × 9 array. Therefore, even in a larger-scale crystal, e.g., a 100 × 100 or infinite array, the excited lattice resonances are expected to appear in the 9 × 9 array area under the same incident beam condition. In contrast, if the beam size is enlarged, we should see different behaviour. For example, the coupling efficiency from the beam to the quadrupole lattice resonances should decrease because the infinite quadrupole lattice resonances under the Bloch boundary condition are purely dark and hence do not allow coupling with free space.

One more important point to discuss is the alignment between the centre of the incident beam and the centre of the crystal or the nanodisk. Interestingly, the alignment scarcely affects the quality of the excited quadrupole lattice resonance. The near-field spectra or field distributions show almost identical profiles for several cases with different beam centre positioning (Supplementary Note 2). This result indicates that the current system provides a robust experimental sample that requires very little alignment precision.

In conclusion, we showed that the quadrupole lattice resonances in a square lattice, finite-sized plasmonic crystal can be excited by vertically incident light beams. Both cylindrical vector beams and a Gaussian beam can excite quadrupole lattice resonances, and the largest peak intensity was obtained in the resonance excited by a specific type of cylindrical vector beam. The combination of the dark mode and lattice resonance leads to lower radiative losses than dipole localized surface plasmon resonances. Our scheme allows for the exploitation of low radiative loss plasmon resonances with many hot spots by using an incident beam from free space. This finding may pave the way for novel light-matter interactions that require a narrow spectral line width or wide field distributions.

## Methods

### Calculation

We numerically calculated the electromagnetic field in our system by using the commercial software COMSOL Multiphysics with RF module. For the infinite periodic system, we performed eigenfrequency calculations with the Bloch boundary condition in the x and y directions and a scattering boundary condition on the top and bottom surfaces of the calculation model. For the finite system, e.g., the 9 × 9 array, we performed harmonic propagation calculation with perfectly matched layers (PMLs) on the side and lower boundaries, plus the scattering boundary condition on the top surface. At the top surface, we excited the incident beam and made it propagate along the –z direction, normal to the plasmonic crystal^16^. The incident beam is defined at the top surface by the three components of the complex electric field (E_x_, E_y_, E_z_) given by vector diffraction theory^31^,^32^.

## Additional Information

**How to cite this article**: Sakai, K. *et al*. Quadrupole lattice resonances in plasmonic crystal excited by cylindrical vector beams. *Sci. Rep.* **6**, 34967; doi: 10.1038/srep34967 (2016).

## Supplementary Material

Supplementary Information
