# Supplementary material for: Quadrupole lattice resonances in plasmonic crystal excited by cylindrical vector beams
Source: Sci Rep. 2016 Oct 13;6:34967. doi: 10.1038/srep34967 (PMC5062317; doi:10.1038/srep34967)
Supplement: Supplementary Information [file srep34967-s1.docx]

SUPPLEMENTARY NOTES

Quadrupole lattice resonances in plasmonic crystal excited by cylindrical vector beams

Kyosuke Sakai*, Kensuke Nomura, Takeaki Yamamoto, Tatsuya Omura, Keiji Sasaki*

Research Institute for Electronic Science, Hokkaido University, Sapporo, Hokkaido, 001-0020 JAPAN, *E-mail: k_sakai@es.hokudai.ac.jp, sasaki@es.hokudai.ac.jp

Supplementary Note 1

**Array size dependence.** The quadrupole lattice resonance is strongly excited by the vertically incident cylindrical vector beam A in the crystals of arbitrary array size, i.e. number (N) of disks on a side. Fig. S1 (a) shows the near field norm (|E|) distributions at the peak wavelength for a series of array sizes; from 3×3 (N=3) to 9×9 (N=9). Fig. S1 (b) shows the corresponding near field spectra taken at the point indicated in the inset of (a). In these array-size regime, where the incident beam is larger than the array, the peak intensity increases as N increases. Fig. S1 (c) shows the peak wavelength and the Full Width at Half Maximum (FWHM) of the near field spectra. Both of them show a converging trend as N increases, indicating that the spectral profile will be identical for even larger crystals under the same incident beam condition. Please note that the arrays with even N, e.g. 2×2, 4×4…, also show similar converging trend in the near field spectrum as N increases, although the beam center penetrate not in the disk center but in the air region.

Fig. S1. Resonance profile for a series of N. (a) Near field norm (|E|) distributions at the peak wavelength. (b) Normarized near field spectra monitored at the point indicated in the inset of (a). (c) Peak wavelength (white circle) and FWHM (black circle) of the normarized near field spectra. For clarity, we drew black curves using are a cubic spline interpolation. The converging trends are clearly seen with increasing N.

Supplementary Note 2

**Placement dependence.** The quadrupole lattice resonances are excited even in the case where the incident beam center deviates from the disk center. Fig. S2 (a) shows the nearfield spectrum and the electric field norm distribution at the peak wavelength for the 8×8 array. The center of the incident beam indicated by the crossing point of two dotted lines penetrates the air region. Fig. S2 (b) shows the other case, in which the 9×9 array is shifted by π, i.e. half of the period, in the y direction. In both cases, the resonant near field spectra show similar profile, i.e. peak at 805 nm with FWHM of ~30 nm. These results indicate that the resonant quality is independent on the placement between the incident beam center and the array center.

Fig. S2. Normalized near field spectrum and near field norm (|E|) distribution at the peak wavelength for (a) 8×8 array and (b) 9×9 array shifted by π in the y direction. The normalized near field spectra are taken at the point depicted by a white point. The cross point of the dotted lines indicates the beam center.
